# Improved Variational Bayes for Space-Time Adaptive Processing

**DOI:** 10.3390/e27030242

**Published:** 2025-02-26

**Authors:** Kun Li, Jinyang Luo, Peng Li, Guisheng Liao, Zhixiang Huang, Lixia Yang

**Affiliations:** 1 School of Electronic Information Engineering, Anhui University, Hefei 230601, China; 2 Sun Create Electronics Co., Ltd., Hefei 230088, China

**Keywords:** space-time adaptive processing, sparse Bayesian learning, variational Bayesian inference, sparse recovery

## Abstract

To tackle the challenge of enhancing moving target detection performance in environments characterized by small sample sizes and non-uniformity, methods rooted in sparse signal reconstruction have been incorporated into Space-Time Adaptive Processing (STAP) algorithms. Given the prominent sparse nature of clutter spectra in the angle-Doppler domain, adopting sparse recovery algorithms has proven to be a feasible approach for accurately estimating high-resolution spatio-temporal two-dimensional clutter spectra. Sparse Bayesian Learning (SBL) is a pivotal tool in sparse signal reconstruction and has been previously utilized, yet it has demonstrated limited success in enhancing sparsity, resulting in insufficient robustness in local fitting. To significantly improve sparsity, this paper introduces a hierarchical Bayesian prior framework and derives iterative parameter update formulas through variational inference techniques. However, this algorithm encounters significant computational hurdles during the parameter update process. To overcome this obstacle, the paper proposes an enhanced Variational Bayesian Inference (VBI) method that leverages prior information on the rank of the temporal clutter covariance matrix to refine the parameter update formulas, thereby significantly reducing computational complexity. Furthermore, this method fully exploits the joint sparsity of the Multiple Measurement Vector (MMV) model to achieve greater sparsity without compromising accuracy, and employs a first-order Taylor expansion to eliminate grid mismatch in the dictionary. The research presented in this paper enhances the moving target detection capabilities of STAP algorithms in complex environments and provides new perspectives and methodologies for the application of sparse signal reconstruction in related fields.

## 1. Introduction

Mounted on elevated platforms, airborne radar exhibits remarkable mobility and effectively addresses the shading limitations of ground-based radar. It provides a substantially longer detection range for ground and low-altitude targets compared to ground-based radar, enjoying superior electromagnetic wave propagation conditions. Consequently, it has garnered considerable attention. Within the diverse applications of airborne radar, moving target detection holds a pivotal position. Phased array radar is frequently employed in airborne systems due to its capacity to generate multiple beams concurrently, its adaptable beam steering, and its robust anti-jamming capabilities. Nevertheless, this radar operates in a downward-facing configuration, where clutter is abundant and intense. Furthermore, the aircraft’s movement results in considerable broadening of the clutter spectrum, frequently causing targets to be obscured within the clutter, significantly hindering the effectiveness of moving target detection [1]. Hence, the foremost objective for airborne radar is clutter suppression. Space-time adaptive processing leverages a combined space-time two-dimensional strategy to devise suitable filter weight vectors, effectively mitigating clutter and preserving target energy across Doppler and angular domains, ultimately bolstering moving target detection performance. STAP technology represents an advancement of array adaptive technology [2]. In 1973, Brennan and Reed pioneered the notion of space-time two-dimensional adaptive processing [3], applying the fundamental principles of array signal processing to the two-dimensional data space of pulse and array element samples, sparking a wave of enthusiasm in STAP research. However, optimal STAP necessitates a substantial quantity of training samples that adhere to the Independent and Identically Distributed (IID) criterion, posing challenges such as elevated computational demands, clutter non-uniformity, and non-stationarity. Consequently, the direct deployment of optimal STAP technology is infeasible.

To mitigate the aforementioned computational demands, recent scholarly endeavors have introduced a diverse array of methodologies. Dimensionality reduction techniques within the space-time adaptive processing framework decrease the system’s degrees of freedom by applying clutter-independent linear transformations. This, in turn, minimizes the requisite number of training samples and lessens computational intricacy [4]. Alternatively, rank reduction STAP methodologies employ clutter-related transformations to fashion adaptive space-time filters, thereby curbing the dependency on extensive training datasets [5,6]. For both dimensionality-reduced and rank-reduced STAP strategies, the needed training samples can be diminished to twice the reduced dimension or clutter rank, respectively; however, in non-uniform settings, the sample size remains considerable. Direct digital domain methodologies [7,8] circumvent the utilization of adjacent range cell training samples by leveraging detection cell data directly to construct the STAP processor. This theoretically tackles all non-uniformity challenges but at the expense of some space-time degrees of freedom and STAP output quality due to the exclusion of samples from other range cells in weight vector formulation. Knowledge-aided STAP approaches augment STAP efficacy by incorporating prior information encompassing environmental conditions, radar system specifics, and platform motion characteristics [9]. The efficacy of this technique heavily leans on the accuracy of the preliminary knowledge. Sparse Recovery STAP (SR-STAP) methodologies exploit the sparsity of clutter distribution in the space-time domain, facilitating the recovery of high-resolution clutter power spectra with limited training samples by estimating the clutter covariance matrix (CCM). This leads to enhanced clutter suppression capabilities. For stationary radar systems, several sparse recovery methods exist, including greedy algorithms, convex optimization techniques, sparse Bayesian learning algorithms, and more. Greedy algorithms iteratively select elements from a predefined set (basis or dictionary) and compute corresponding sparse coefficients, progressively minimizing the discrepancy between the linear combination of these elements and the observed data [10]. Common greedy algorithms encompass Matching Pursuit [11], Orthogonal Matching Pursuit [12], Relaxed Greedy Algorithm [13], and $L_{1}$-norm Greedy Algorithm, among others. These algorithms are advantageous for their flexibility in incorporating constraints [12]; however, they may yield suboptimal sparse coefficient solutions in certain scenarios [13]. Convex optimization methods recast the optimization challenge into a convex framework and solve for the sparse coefficient vector by leveraging the properties of convex functions. Prominent convex optimization algorithms include interior point methods and gradient descent-based approaches. Interior point methods, being among the earliest convex optimization techniques for sparse problems, are mature and accompanied by readily accessible software tools. They are sensitive to solution sparsity and regularization parameters but suffer from high computational complexity, especially for high-dimensional signals. Gradient descent-based algorithms, such as Iterative Splitting and Thresholding (ITS) [14], Two-step IST (TwIST), Fixed-Point Iteration (FPI), and others, closely depend on the regularization parameter’s magnitude. The burgeoning SBL approach, introduced by Tipping around 2001 [15], assumes a sparse prior distribution for the coefficient vector and employs a maximum posterior estimator to integrate prior knowledge with observations for sparse vector recovery. In noiseless conditions, SBL yields the most accurate sparse solution and maintains robustness even when the sensing matrix columns are highly correlated. However, Bayesian learning entails matrix inversion in each iteration, substantially augmenting computational complexity and posing challenges for real-time applications [16]. In recent years, the application of Bayesian methods in the field of communications has become increasingly widespread, particularly showcasing its unique advantages in channel estimation and signal processing. By incorporating prior information, Bayesian methods can effectively handle the uncertainty and sparsity in high-dimensional data, thereby enhancing estimation accuracy and computational efficiency. For example, Cheng et al. [17] proposed a Bayesian channel estimation algorithm based on irregular array manifolds, which transforms the channel estimation problem into a tensor decomposition problem under missing data scenarios and incorporates Bayesian model order selection techniques to automatically estimate the number of channel paths and significantly improve estimation accuracy. Xu et al. [18] introduced a Bayesian multiband sparsity-based channel estimation framework to address the beam squint effect in millimeter-wave massive MIMO systems. By constructing a virtual channel model and leveraging the common sparse structure across sub-bands, combined with a first-order Taylor expansion to mitigate dictionary grid mismatch, their variational Expectation-Maximization (EM) algorithm can adaptively balance the likelihood function and sparse prior information, significantly enhancing channel estimation accuracy in dual-broadband scenarios. These works highlight the unique advantages of Bayesian methods in joint sparse modeling and computational efficiency optimization, providing important insights for clutter spectrum estimation in STAP.

The SR-STAP based on Bayesian learning necessitates matrix inversion during each iteration, resulting in a steep surge in computational complexity as the system’s dimensionality expands [19]. To mitigate this challenge, various prominent strategies have been proposed. Specifically, a rapid inversion-free approach for Sparse Bayesian Learning (SBL) was presented in [19], leveraging the fact that inverting diagonal matrices is significantly faster than traditional matrix inversion. Nonetheless, this method may suffer from performance decrement in scenarios with a relatively limited number of measurements, stemming from the relaxation of the evidence bound. In another study [20], the Spatial Alternating Variational Estimation (SAVE) method was introduced, which circumvents matrix updates by alternately optimizing each signal element. This approach significantly accelerates the reconstruction process for signals characterized by small-dimensional samples. However, its efficacy diminishes when dealing with signals of exceptionally high dimensionality. Furthermore, Al-Shoukairi et al. [21] put forward an SBL algorithm based on Generalized Approximate Message Passing (GAMP). By employing quadratic approximations and Taylor series expansions, GAMP furnishes approximations for the Maximum A Posteriori (MAP) estimates of the signal, thereby bypassing the need for matrix inversion. Nevertheless, the introduction of an iterative method to replace matrix inversion in SBL still fails to substantially alleviate the computational strain associated with large-scale datasets [22]. To address the limitations of existing methods, we introduce a novel approach that capitalizes on prior knowledge concerning the rank of the space-time clutter covariance matrix. Our contribution lies in an enhanced Variational Bayesian method, which optimizes the parameter update formulations to circumvent the inefficiencies associated with high-dimensional matrix inversion, thereby effectively minimizing computational complexity.

## 2. Signal Model

**Figure 1:**
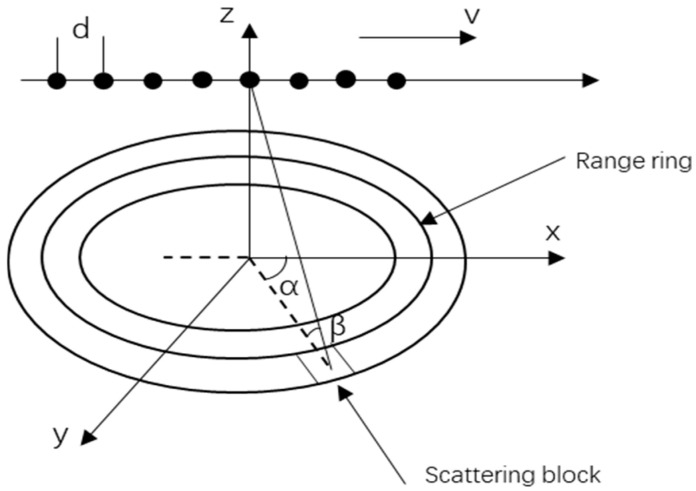
Schematic diagram of airborne radar.

Consider an airborne side-looking uniform linear array (ULA) radar comprised of N array elements, with each element spaced at a distance d, equivalent to half the radar’s operational wavelength. The height of the carrier platform is designated as H, the frequency of pulse repetition is expressed as $f_{r}$, and the count of pulses within the coherent processing interval (CPI) is indicated by M. The geometrical representation of this airborne radar setup is depicted in Figure 1.

Considering v as the velocity of the carrier platform traversing along the x-axis, and α and β signifying the elevation and azimuth angles of the ground reflection point, respectively, the t-th range ring’s space-time snapshot data, incorporating both clutter and noise, and disregarding the impact of range ambiguity, can be formulated as follows:

(1) $$x\left(t\right)=\sum_{i=1}^{N_{c}}\delta_{i}V(f_{d,i},f_{s,i})+n_{0}$$

In the formula, $N_{c}$ denotes the count of clutter patches contained within the specific range bin, while $\delta_{i}$ indicates the scattering power of each individual clutter patch. $n_{0}$, which belongs to the complex space $C^{\mathrm{NM}\times 1}$, represents the thermal noise component. Furthermore, ${V(f}_{d,i},f_{s,i})$ stands for the space-time steering vector associated with the i-th clutter patch and can be formulated as follows:

(2) $${V(f}_{d,i},f_{s,i})=V_{d}\left(f_{d,i}\right)⨂V_{s}\left(f_{s,i}\right)$$

While

(3) $$V_{d}(f_{d,i})={\left[1,e^{j2\pi f_{d,i}},\cdots ,e^{j2\pi \left(M-1\right)f_{d,i}}\right]}^{T}$$

(4) $$V_{s}(f_{s,i})={\left[1,e^{j2\pi f_{s,i}},\cdots ,e^{j2\pi \left(N-1\right)f_{s,i}}\right]}^{T}$$

In the formula, ⨂ is used to indicate the Kronecker product, while ${\left[\cdot \right]}^{T}$ stands for the transposition of a matrix. $f_{d,i}$ and $f_{s,i}$ represent the normalized Doppler frequency and normalized spatial frequency, respectively, corresponding to the i-th clutter patch, and they can be formulated as outlined below:

(5) $$f_{d,i}=\frac{2\mathrm{vcos}\alpha_{i}\cos\beta_{i}}{\lambda f_{r}}$$

(6) $$f_{s,i}=\frac{\mathrm{dcos}\alpha_{i}\cos\beta_{i}}{\lambda }$$

In the equation, λ represents the wavelength.

Assuming that the snapshot data of each range bin are independent and identically distributed (IID), the covariance matrix can be expressed as follows [23]:

(7) $$R_{C}=E[x(t)x^{H}\left(t\right)]$$

In the equation, E[·] denotes the mathematical expectation operation, and ${\left(\cdot \right)}^{H}$ represents the conjugate transpose of a matrix.

Based on the Linearly Constrained Minimum Variance (LCMV) criterion, the optimal STAP weight vector can be expressed as follows:

(8) $$W_{\mathrm{OPT}}=\frac{{R_{C}}^{-1}V_{t}\left(f_{d},f_{s}\right)}{{V_{t}}^{H}\left(f_{d},f_{s}\right){R_{C}}^{-1}V_{t}\left(f_{d},f_{s}\right)}$$

In the equation, ${\left(\cdot \right)}^{-1}$ denotes the matrix inversion operation, and $V_{t}(f_{d,t},f_{s,t})$ represents the space-time steering vector of the target, which can be expressed as follows:

(9) $$V_{t}(f_{d,t},f_{s,t})=V_{d}(f_{d,t})⨂V_{s}(f_{s,t})$$

While

(10) $$V_{d}(f_{d,t})={\left[1,e^{j2\pi f_{d,t}},\cdots ,e^{j2\pi \left(M-1\right)f_{d,t}}\right]}^{T}$$

(11) $$V_{s}(f_{s,t})={\left[1,e^{j2\pi f_{s,t}},\cdots ,e^{j2\pi \left(N-1\right)f_{s,t}}\right]}^{T}$$

While

(12) $$f_{d,t}=\frac{2v_{t}\cos\alpha_{t}\cos\beta_{t}}{\lambda }$$

(13) $$f_{s,t}=\frac{\mathrm{dcos}\alpha_{t}\cos\beta_{t}}{\lambda }$$

In the equation, $f_{d,t}$ and $f_{s,t}$ represent the Doppler normalized frequency and spatial normalized frequency of the target, respectively, while $\alpha_{t}$ and $\beta_{t}$ signify the elevation angle and azimuth angle of the target, respectively.

Finally, the filtered snapshot data is represented as follows:

(14) $$s(t)={W_{\mathrm{OPT}}}^{H}x(t)$$

Given that the Doppler and spatial frequencies of the clutter space-time snapshot signals outlined in Equation (1) are confined within a specific range, a comprehensive set of these frequencies can be derived using an exhaustive approach, with a tolerance for a certain degree of quantization error. This set is denoted as follows:

(15) $$\Phi_{0}=[V(f_{d,1},f_{s,1}),V(f_{d,1},f_{s,2}),\cdots ,V(f_{d,N_{s}},f_{s,N_{s}})]$$

where $N_{d}=\rho_{d}N$ and $N_{S}=\rho_{s}M$, with $\rho_{d}$ >> 1 and $\rho_{s}$ >> 1 indicating the resolution scales for the Doppler frequencies and spatial frequencies, respectively. These resolution scales serve to regulate the extent of quantization error. Consequently, the space-time snapshot data pertaining to the clutter in the Equation (1) can alternatively be formulated as:

(16) $$x(t)=\sum_{k=1}^{N_{d}}\sum_{i=1}^{N_{s}}\delta_{k,i}V(f_{d,k},f_{s,i})+n_{0}=\Phi_{0}y(t)+n_{0}$$

In the equation, $y(t)={[y_{1,1}(t),y_{1,2}(t),\cdots ,y_{N_{d},N_{s}}\left(t)\right]}^{T}$ represents the complex amplitude of the clutter space-time snapshot data x(t) on the angle-Doppler image, which can also be referred to as the angle-Doppler image.

Considering the space-time steering dictionary set in Equation (15), its column vectors correspond to discretized normalized Doppler frequencies $f_{d}$ and spatial frequencies $f_{s}$. The actual frequencies $\left(\tilde{f_{d}},\tilde{f_{s}}\right)$ of clutter scatterers may deviate from the predefined grid points. To address this, grid offsets $\delta_{d}\in R^{N_{d}}$ and $\delta_{s}\in R^{N_{s}}$ are introduced to model the true frequencies as: $\tilde{f_{d}}=f_{d}+\delta_{d},\tilde{f_{s}}=f_{s}+\delta_{s}$. During initialization, set all elements of both “δ_d” and “δ_s” to 0. Using a first-order Taylor expansion, the off-grid steering vector can be approximated as:

(17) $$V(\tilde{f_{d}},\tilde{f_{s}})=V(f_{d},f_{s})+\frac{\partial V}{\partial f_{d}}\delta_{d}+\frac{\partial V}{\partial f_{s}}\delta_{s}$$

In the equation, the partial derivative terms are:

$$\frac{\partial V}{\partial f_{d}}=j2\pi \cdot \mathrm{diag}(\left[0,1,\cdots ,M-1\right])V(f_{d},f_{s})$$

$$\frac{\partial V}{\partial f_{s}}=j2\pi \cdot \mathrm{diag}(\left[0,1,\cdots ,N-1\right])V(f_{d},f_{s})$$

Therefore, the off-grid dictionary set can be rewritten as follows:

(18) $$\Phi =\Phi_{0}+\Phi_{d}\mathrm{diag}\left(\delta_{d}\right)+\Phi_{s}\mathrm{diag}\left(\delta_{s}\right)$$

In the equation, $\Phi_{d}$ and $\Phi_{s}$ represent the Jacobians of $\Phi_{0}$ with respect to $f_{d}$ and $f_{s}$, respectively.

Then, Equation (7) can be re-expressed as:

(19) $$R_{C}=E[x(t)x^{H}\left(t\right)]=\Phi^{H}\mathrm{diag}(P)\Phi +\sigma_{n}^{2}I_{\mathrm{NM}}$$

In the equation, $\sigma_{n}^{2}$ is the noise power, $I_{\mathrm{NM}}$ is an identity matrix of dimension NM × NM; $P={\left[p_{1,1,}p_{1,2,}\cdots p_{N_{d},N_{s}}\right]}^{T}$, where $p_{k,i}=E[{\left|\delta_{k,i}\right|}^{2}]$, for $k=1,2,\cdots N_{d}$ and $i=1,2,\cdots N_{s}$.

Similarly, for the Multiple Measurement Vector (MMV) scenario, the following equation applies:

(20) $$X=\Phi Y+N_{0}$$

In the equation, $X=[x\left(1\right),x\left(2\right),\cdots ,x\left(L\right)]$ and $Y=[y\left(1\right),y\left(2\right),\cdots ,y\left(L\right)]$, where L represents the number of snapshot data points, and each column of $N_{0}$ denotes the received Gaussian white noise.

Assuming that the training samples satisfy the IID condition, the implementation for solving the sparse solution using the MMV method can be expressed as follows:

(21) $$Y=\min{\left||Y|\right|}_{2,0},s.t.{\left||X-\Phi Y|\right|}_{F}^{2}$$

In the equation, ${\left||\cdot |\right|}_{2,0}$ is a mixed norm defined as the number of zero elements in the vector formed by the $L_{2}$ norms of each row vector. ${\left||\cdot |\right|}_{F}$ denotes the Frobenius norm.

In Equation (21), the involvement of the $L_{0}$ norm has proven that solving the aforementioned optimization problem is NP-hard. In pursuit of sparsity, dealing with optimization problems and requiring mathematical approximations, the $L_{1}$ norm can be used as a substitute for the $L_{0}$ norm. Therefore, the above equation can be rewritten as:

(22) $$Y=\min{\left||Y|\right|}_{2,1},s.t.{\left||X-\Phi Y|\right|}_{F}^{2}$$

In the equation, ${\left||\cdot |\right|}_{2,1}$ is a mixed norm defined as the $L_{1}$ norm of the vector formed by the $L_{2}$ norms of each row vector.

## 3. The Proposed Method

### 3.1. Bayesian Framework

Constructing an apt Bayesian model is indispensable and pivotal for SBL. In this paper, we incorporate hierarchical prior information into the latent variables to further augment sparsity. The likelihood associated with the observed variable X is expressed as follows:

(23) $$p(X|Y,\sigma ,\delta_{d},\delta_{s})=\prod_{t=1}^{L}\mathrm{CN}(\Phi y(t),\sigma^{-1}I_{\mathrm{NM}})$$

In the equation, σ represents the noise precision.

By imposing a Gaussian distribution prior on the latent variable Y, we obtain:

(24) $$p(Y|\gamma )=\prod_{t=1}^{L}\mathrm{CN}\left(y\left(t\right)|0,\Sigma \right)$$

**Figure 2:**
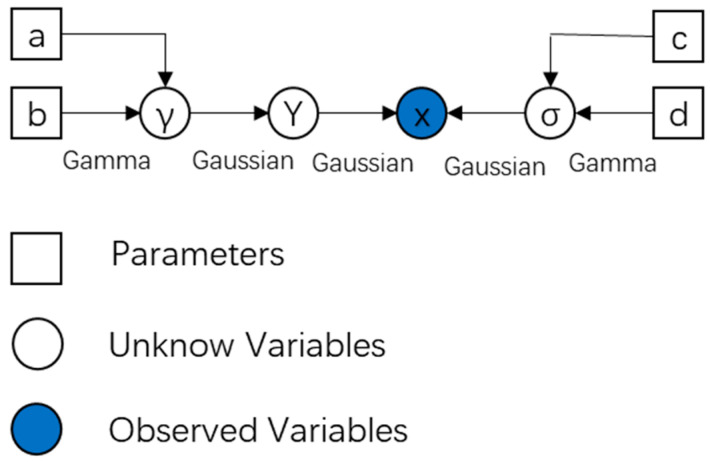
Directed acyclic graph of the proposed Bayesian model.

In the equation, $\gamma =[\gamma_{1},\gamma_{2},\gamma_{N_{d}N_{s}}]$ and Σ=diag(γ). Since the inverse Gamma distribution is conjugate to the Gaussian distribution, we adopt a Gamma distribution prior for each element of γ, which can be expressed as:

(25) $$p(\gamma |a,b)=\prod_{m=1}^{N_{d}N_{s}}\frac{b^{a}\gamma_{m}^{a-1}e^{-b\gamma_{m}}}{\Gamma \left(a\right)}$$

where $\Gamma (a)=\int_{0}^{+\infty }x^{a-1}e^{-x}\mathrm{dx}$, with a being the shape parameter and b being the scale parameter. Similarly, assuming that σ follows a Gamma distribution, we obtain:

(26) $$p(\sigma |c,d)=\frac{c^{d}\sigma^{c-1}e^{-d\sigma }}{\Gamma \left(c\right)}$$

where c and d are the corresponding shape and scale parameters, respectively. The directed acyclic graph for representing the Bayesian model is shown in Figure 2.

Based on the aforementioned Bayesian hierarchical model assumptions, the joint distribution of the signals can be obtained as:

(27) $$p(X,Y,\gamma ,\sigma |\delta_{d},\delta_{s})=p(X|Y,\sigma ,\delta_{d},\delta_{s})p(Y|\gamma )p(\gamma |a,b)p(\sigma |c,d)$$

### 3.2. Minimization of KL Divergence (Variational E-Step)

According to Bayesian theory, the maximum posterior probability of the parameters to be estimated can be obtained, but its calculation usually involves high-dimensional and complex integrals, making it difficult to solve. Therefore, we introduce variational inference to address this issue of maximum posterior estimation.

In variational inference, the observed data X represents the data received by the array elements, and the set of latent variables ξ={Y,γ,σ} consists of the parameters to be estimated. The following equation holds:

(28) $$\ln p(X|\delta_{d},\delta_{s})=L(q)+\mathrm{KL}(q||p)=\int q\left(\xi \right)\ln\left\{\frac{p\left(X,\xi \right)}{q\left(\xi \right)}\right\}d\xi -\int q(\xi )\ln\{\frac{p\left(\xi |X\right)}{q\left(\xi \right)}\}d\xi$$

In the equation, L(q) represents the Evidence Lower Bound (ELBO), and KL(q||p) denotes the Kullback-Leibler (KL) divergence, which measures the approximation degree between the probability distribution q and the posterior distribution p. The smaller the KL divergence, the higher the degree of approximation. The goal of variational inference is to maximize the lower bound of ln⁡p(X) by finding the distribution of q(ξ) that maximizes the ELBO. At this point, q(ξ) can be used to approximate the posterior probability distribution of the latent variables.

To minimize KL Divergence, the probability distribution is decomposed into q(ξ)=q(Y)q(γ)q(σ), and the general expression for the optimal approximate distribution is provided as follows:

(29) $$\ln q_{j}^{*}(\xi_{j})=E_{i\neq j}[\ln p(X,\xi |\delta_{d},\delta_{s})]+\mathrm{const}$$

In the expression, $E_{i\neq j}\left[\cdot \right]$ denotes the conditional expectation of the parameter $\xi_{i}$ under the approximate distribution q, with the condition that $\xi_{i}$ for i≠j is held fixed.

By ignoring the terms unrelated to Y, the optimal approximate posterior distribution for q(Y) can be obtained from Equation (29) as follows:

(30) $$q^{*}\left(Y\right)={<\ln p\left(X|Y,\rho ,\delta_{d},\delta_{s}\right)p\left(Y|\gamma \right)>}_{q\left(\gamma \right)q\left(\rho \right)}+\mathrm{const}$$

where ${<\cdot >}_{q\left(\cdot \right)}$ denotes the expectation concerning $q\left(\cdot \right)$, then $q^{*}(Y)$ can be solved as being subject to a joint complex Gaussian distribution, which is expressed as:

(31) $$q^{*}\left(Y\right)=\prod_{t=1}^{L}\mathrm{CN}(y(t);\mu (t),\Sigma_{Y})$$

While

(32) $$\mu (t)=\rho \Sigma_{Y}\Phi^{H}x(t)$$

(33) $$\Sigma_{Y}={\left(\rho \Phi^{H}\Phi +\Sigma^{-1}\right)}^{-1}$$

Ignoring the terms unrelated to γ, the optimal approximate posterior distribution for q(γ) can be obtained from Equation (29) as follows:

(34) $$q^{*}\left(\gamma \right)={<\ln p\left(Y|\gamma \right)p\left(\gamma |a,b\right)>}_{q\left(Y\right)q\left(\rho \right)}+\mathrm{const}$$

$q^{*}(\gamma )$ is solved to be a Gamma distribution, with its probability distribution expressed as:

(35) $$q^{*}\left(\gamma \right)=\prod_{m=1}^{N_{d}N_{s}}\Gamma (\gamma_{m};a+\frac{1}{2}L,b+b_{m}^{\gamma })$$

(36) $${\gamma_{m}}^{\mathrm{new}}={<\gamma_{m}>}_{q(\gamma_{m})}=\frac{a+\frac{1}{2}L}{b+b_{m}^{\gamma }}$$

In the equation, $b_{m}^{\gamma }=\frac{1}{2}\sum_{t=1}^{L}[({\mu_{m}\left(t)\right)}^{2}+{\Sigma_{Y}}_{m,m}]$, where $\mu_{m}\left(t\right)$ denotes the m-th element of the vector μ(t), and $\Sigma_{Y}$ represents the element in the m-th row and m-th column of the matrix $\Sigma_{Y}$.

Ignoring the terms unrelated to σ, the optimal approximate posterior distribution for q(σ) can be obtained from Equation (29) as follows:

(37) $$q^{*}\left(\rho \right)={<\ln p\left(X|Y;\rho \right)p\left(\rho |c,d\right)>}_{q\left(Y\right)q\left(\gamma \right)}+\mathrm{const}$$

$q^{*}\left(\rho \right)$ is solved to be a Gamma distribution, with its probability distribution stated as follows:

(38) $$q^{*}\left(\rho \right)=\Gamma (\rho ;c+\frac{1}{2}\mathrm{LNM},d+d_{\rho })$$

(39) $$\rho^{\mathrm{new}}={<\rho >}_{q(\rho )}=\frac{c+\frac{1}{2}\mathrm{LNM}}{d+d_{\rho }}$$

In the equation, $d_{\rho }=\frac{1}{2}{||X-\Phi U||}_{2}^{2}+\frac{1}{2}\mathrm{tr}(\Phi^{H}\Sigma_{Y}\Phi )$, where $U=[\mu \left(1\right),\mu \left(2\right),\cdots ,\mu \left(L\right)]$ and tr(·) denotes the trace of a matrix.

### 3.3. Maximization of the Lower Bound (M-Step)

When q(ξ) is fixed, the maximization of the lower bound of Equation (28) is:

(40) $$\max_{\delta_{d},\delta_{s}}L\left(\delta_{d},\delta_{s}\right)={<\ln p(X,\xi |\delta_{d},\delta_{s})\;>}_{q(\xi )}+\mathrm{const}$$

Ignoring the terms unrelated to $\delta_{d}$ and $\delta_{s}$, Equation (40) can be simplified as [18]:

(41) $$\min_{\delta_{d},\delta_{s}}\{-2R\left(v_{d}^{T}\delta_{d}+v_{s}^{T}\delta_{s}\right)+\delta_{d}^{T}E_{\mathrm{dd}}\delta_{d}+\delta_{s}^{T}E_{\mathrm{ss}}\delta_{s}+2\delta_{d}^{T}R\left(E_{\mathrm{dS}}\right)\delta_{s}\}$$

In the equation, R(∙) denotes taking the real part, where:

$$v_{d}=\sum_{t=1}^{L}\mathrm{diag}(\mu (t))\Phi_{d}^{T}E[x\left(t\right)-\Phi \mu (t)]$$

$$v_{s}=\sum_{t=1}^{L}\mathrm{diag}(\mu \left(t)\right)\Phi_{s}^{T}E[x\left(t\right)-\Phi \mu \left(t)\right]$$

$$E_{\mathrm{dd}}=\sum_{t=1}^{L}\mathrm{diag}(\mu \left(t)\right)\Phi_{d}^{T}\Phi_{d}\mathrm{diag}(\mu (t))+\mathrm{tr}(\Phi_{d}^{T}\Phi_{d}\Sigma_{Y})$$

$$E_{\mathrm{ss}}=\sum_{t=1}^{L}\mathrm{diag}(\mu \left(t)\right)\Phi_{s}^{T}\Phi_{s}\mathrm{diag}(\mu (t))+\mathrm{tr}(\Phi_{s}^{T}\Phi_{s}\Sigma_{Y})$$

$$E_{\mathrm{ds}}=\sum_{t=1}^{L}\mathrm{diag}(\mu \left(t)\right)\Phi_{d}^{T}\Phi_{s}\mathrm{diag}(\mu (t))+\mathrm{tr}(\Phi_{d}^{T}\Phi_{s}\Sigma_{Y})$$

Then, by setting the derivatives of Equation (41) with respect to $\delta_{d}$ and $\delta_{s}$ to zero, the optimal solution for Equation (41) can be obtained.

(42) $$\left[\begin{array}{c} {\delta_{d}}^{\mathrm{new}}\\ {\delta_{s}}^{\mathrm{new}} \end{array}\right]={\left[\begin{array}{cc} E_{\mathrm{dd}} & R\left(E_{\mathrm{dS}}\right)\\ {R\left(E_{\mathrm{dS}}\right)}^{T} & E_{\mathrm{ss}} \end{array}\right]}^{-1}\left[\begin{array}{c} {R(v}_{d})\\ R(v_{d}) \end{array}\right]$$

The iterative algorithm for Variational Bayesian Inference is structurally simple, with the specific steps detailed in Algorithm 1.
**Algorithm 1.** VB-SR-STAP algorithms.Stap1:$\mathrm{Set}\;\mathrm{initial}\;\mathrm{values}\;\mathrm{for}\;\mathrm{hyperparameters}\;\gamma_{0}$$,\;\mathrm{noise}\;\mathrm{precision}\;\sigma_{0}$, a predefined error tolerance ε$,\;\mathrm{maximum}\;\mathrm{iteration}\;\mathrm{count}\;k_{\max}$$,\;\mathrm{and}\;\mathrm{parameters}\;a,b,c,d,\delta_{d},\delta_{s}$Stap2:Use Equations (32) and (33) to update μ(t)$\mathrm{and}\;\Sigma_{Y},$ respectivelyStap3:Use Equations (36) and (39) to update γand σ, respectivelyStap4:$\mathrm{Use}\;\mathrm{Equation}\;(42)\;\mathrm{to}\;\mathrm{update}\;\delta_{d}$$\mathrm{and}\;\delta_{s}$, and use Equation (18) to update ΦStap5:Increase k by 1Stap6:$\mathrm{If}\;\frac{{\left|\right|\gamma^{\mathrm{new}}-\gamma^{\mathrm{old}}\left|\right|}_{2}}{{\left|\right|\gamma^{\mathrm{old}}\left|\right|}_{2}}<\varepsilon$$\mathrm{or}\;k>k_{\max}$, then return to Step 2Stap7:Output the final result of μ(t)Stap8:$\mathrm{First}\;\mathrm{calculate}\;R_{C}=\frac{1}{L}\sum_{t=1}^{L}[\left(\Phi \mu (t)\right){\left(\Phi \mu (t)\right)}^{H}]$$,\;\mathrm{and}\;\mathrm{subsequently}\;\mathrm{calculate}\;w_{\mathrm{OPT}}=\frac{{R_{C}}^{-1}V_{t}(f_{d},f_{s})}{{V_{t}}^{H}(f_{d},f_{s}){R_{C}}^{-1}V_{t}(f_{d},f_{s})}$

### 3.4. Improved Variational Bayesian Inference

For a squint-looking uniform linear array Doppler pulse radar, assuming a constant pulse repetition frequency, constant platform velocity, and idealized clutter conditions where the clutter scatterers are stationary with no internal motion, the clutter subspace can be approximated by a subspace computed using a set of space-time steering vectors. These space-time steering vectors satisfy the following conditions:

(43) $$\bar{V_{p}\;}={\left[1,\cdots ,e^{j2\pi f_{s}^{p}\left(\beta n+m\right),\cdots ,}e^{j2\pi f_{s}^{p}\left(\beta \left(N-1\right)+M-1\right)}\right]}^{T}$$

While

$$f_{s}^{p}=\frac{p}{N_{r}},p=0,1,\cdots N_{r}-1$$

$$\beta =\frac{f_{d}}{f_{s}}=\frac{2v}{df_{r}}$$

In the equation, $N_{r}$ represents the rank of the clutter, which can be estimated using the well-known Brennan’s rule [23]. When $\beta =\frac{2v}{df_{r}}$ is an integer less than M, the rank of the clutter covariance matrix satisfies the following equation:

(44) $$\mathrm{Rank}(R_{C})=N_{r}=\beta (N-1)+M$$

When $\beta =\frac{2v}{df_{r}}$ is a decimal number less than M, the rank of the clutter covariance matrix satisfies the following relationship:

(45) $$\mathrm{Rank}(R_{C})\approx N_{r}=\lfloor \beta (N-1)+M\rfloor$$

In the formula, ⌊∙⌋ denotes the floor function. Then, the t-th space-time snapshot data in Equation (1) can also be expressed as the following equation:

(46) $$x(t)=\sum_{p=0}^{N_{r}}\bar{\delta_{p}}\bar{V_{p}}$$

In the equation, $\bar{\delta_{p}}$, where $p=1,2,\cdots ,N_{r}-1$, are complex coefficients corresponding to the space-time steering vectors $\bar{V_{p}}$. When the space-time steering dictionary includes all space-time steering vectors ${\left\{\bar{V_{p}}\right\}}_{p=0}^{N_{r}-1}$, the clutter space-time snapshot data represented by Equation (46) can also be expressed in the form of Equation (16). At this point, the non-zero elements in the angle-Doppler image correspond to the complex coefficients $\bar{\delta_{p}}$, where $p=1,2,\cdots ,N_{r}-1$. This indicates that the number of non-zero elements in the clutter-Doppler spectrum, $N_{r}$, can be much smaller than the number of clutter scatterers, $N_{c}$. Furthermore, the space-time snapshot data of the clutter can be fully recovered using only $N_{r}$ space-time steering vectors from the dictionary.

The previous analysis shows that μ(t) is a highly sparse signal, with most of its terms being close to zero. Additionally, Equation (36) indicates that when a corresponding term $\mu_{m}\left(t\right)$ in μ(t) is non-zero, due to the typically very small settings of parameters a and b, the corresponding term $\gamma_{m}$ will be extremely small. Conversely, if a certain term $\gamma_{m}$ of γ is large, the probability that the corresponding term $\mu_{m}\left(t\right)$ in μ(t) is zero is high. Since each term of μ(t) corresponds to a complex coefficient of a space-time steering vector in the dictionary set, $\mu_{m}\left(t\right)$ is non-zero only when clutter exists at the corresponding angle-Doppler bin. Therefore, when $\gamma_{m}$ is small, the corresponding $\mu_{m}\left(t\right)$ (clutter in the angle-Doppler image) is non-zero. Furthermore, based on the aforementioned prior knowledge that there are approximately $N_{r}$ non-zero solutions in μ(t), and inspired by a K-means clustering sparse Bayesian learning algorithm proposed in the literature [24], as well as the rank pruning techniques presented in references [17,18], combining these information allows us to significantly reduce the number of iterations by only updating the values of μ(t) corresponding to the $N_{r}$ smallest values of γ. The update rules for each iteration can be stated as follows: (1) Record the indices of the $N_{r}$ smallest elements in the hyperparameter γ to form a heap set A; (2) Update the terms in μ(t) whose indices are in the heap set A. The corresponding update formulas then become as follows:

(47) $$\mu_{m}\left(t\right)=\rho \sigma_{m}^{2}\Phi_{m}^{H}x(t)$$

(48) $$\sigma_{m}^{2}={\left(\rho \Phi_{m}^{H}\Phi_{m}+{\gamma_{m}}^{-1}\right)}^{-1}$$

where $\Phi_{m}$ denotes the m-th column of matrix Φ, and $\mu_{m}\left(t\right)$ and $\sigma_{m}^{2}$ represent the mean and variance of the complex coefficient corresponding to the m-th space-time steering vector, respectively. The specific steps of the improved iterative algorithm are listed in Algorithm 2.
**Algorithm 2.** IVB-SR-STAP algorithms.Stap1:$\mathrm{Set}\;\mathrm{initial}\;\mathrm{values}\;\mathrm{for}\;\mathrm{hyperparameters}\;\gamma_{0}$$,\;\mathrm{noise}\;\mathrm{precision}\;\sigma_{0}$, a predefined error tolerance ε$,\;\mathrm{maximum}\;\mathrm{iteration}\;\mathrm{count}\;k_{\max}$, and parameters a,b,c,d$,\delta_{d},\delta_{s}$Stap2:$\mathrm{Update}\;\mathrm{all}\;\mathrm{elements}\;\mathrm{of}\;\mu (t)\;\mathrm{and}\;\Sigma_{Y}$$\mathrm{using}\;\mathrm{Equations}\;(32)\;\mathrm{and}\;(33),\;\mathrm{respectively},\;\mathrm{and}\;\mathrm{identify}\;\mathrm{the}\;\mathrm{indices}\;\mathrm{of}\;\mathrm{the}\;N_{r}$smallest elements in γ to form the initial heap set AStap3:$\mathrm{If}\;\mathrm{the}\;\mathrm{index}\;m\;\mathrm{of}\;\gamma_{m}$belongs to A$,\;\mathrm{update}\;\sigma_{m}^{2}$$\mathrm{using}\;\mathrm{Equation}\;(48)\;\mathrm{and}\;\mathrm{the}\;\mathrm{corresponding}\;\mu_{m}\left(t\right)$ using Equation (47)Stap4:Use Equations (36) and (39) to update γand σ, respectivelyStap5:Update set AStap6:$\mathrm{Use}\;\mathrm{Equation}\;(42)\;\mathrm{to}\;\mathrm{update}\;\delta_{d}$$\mathrm{and}\;\delta_{s}$, and use Equation (18) to update ΦStap7:Increase k by 1Stap8:$\mathrm{If}\;\frac{{\left|\right|\gamma^{\mathrm{new}}-\gamma^{\mathrm{old}}\left|\right|}_{2}}{{\left|\right|\gamma^{\mathrm{old}}\left|\right|}_{2}}<\varepsilon$$\mathrm{or}\;k>k_{\max}$, then return to Step 3Stap9:Output the final result of μ(t)Stap10:$\mathrm{First}\;\mathrm{calculate}\;R_{C}=\frac{1}{L}\sum_{t=1}^{L}[\left(\Phi \mu (t)\right){\left(\Phi \mu (t)\right)}^{H}]$$,\;\mathrm{and}\;\mathrm{subsequently}\;\mathrm{calculate}\;w_{\mathrm{OPT}}=\frac{{R_{C}}^{-1}V_{t}(f_{d},f_{s})}{{V_{t}}^{H}(f_{d},f_{s}){R_{C}}^{-1}V_{t}(f_{d},f_{s})}$

### 3.5. Comparison of Computational Complexity

The main focus of this article is on the frequency of multiplication and division operations during a single iteration of an algorithm, and it conducts an in-depth analysis of its computational complexity. The proposed algorithm exhibits a computational complexity of $O(ZN_{r})$ when calculating the variables μ(t) and $\Sigma_{Y}$, where Z=MN. Meanwhile, the computational complexity for calculating the variable γ is $O(N_{r})$. As a result, the total computational complexity per iteration amounts to $O(ZN_{r})$. In comparison, the computational complexities reported in the pieces of literature [15,20] are O(Z$\tilde{Z}$^2^) and O(Z$\tilde{Z}$). Where $\tilde{Z}=N_{S}N_{d}>N_{r}$, it is evident that the proposed algorithm significantly reduces computational complexity.

## 4. Experimental Simulation

### 4.1. Analysis of Clutter Power Spectrum

The performance of the IVB-SR-STAP algorithms was analyzed through simulation experiments, and compared with the Homotopy-SR-STAP [25], LMSSE-SR-STAP [26], and VB-SA-STAP algorithms [20]. Table 1 presents the simulation parameters for a radar system configured with a uniformly spaced linear array in broadside-looking configuration.

#### 4.1.1. Clutter Power Spectrum Under Ideal Conditions

**Figure 3:**
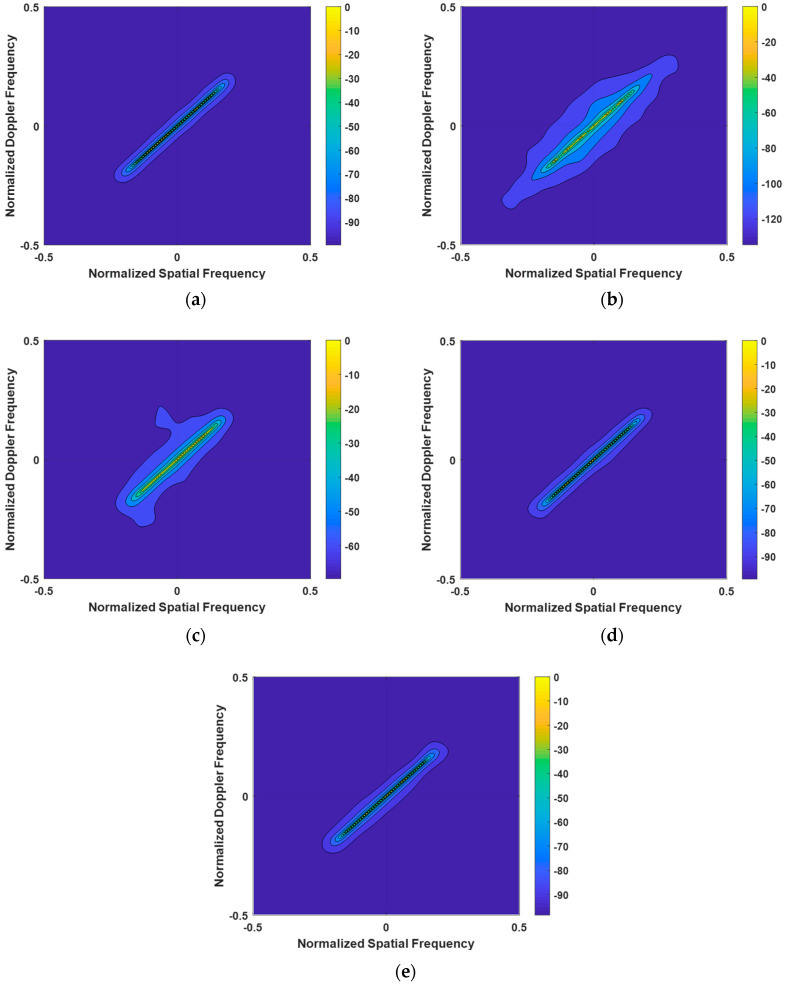
Clutter Power Spectrum under Ideal Conditions. (**a**) OPT; (**b**) LSSME-SR-STAP algorithm; (**c**) Homotopy-SR-STAP algorithm; (**d**) VB-SR-STAP algorithm; (**e**) IVB-SR-STAP algorithm.

The first experiment meticulously examined the clutter power spectra of the LMSSE-SR-STAP, Homotopy-SR-STAP, VB-SR-STAP, and IVB-SR-STAP algorithms under ideal conditions, with the results presented in Figure 3. The LMSSE-SR-STAP and Homotopy-SR-STAP algorithms require the setting of regularization parameters; however, it is difficult to determine an optimal value for these parameters. This has led to a noticeable broadening phenomenon in the clutter spectrum, resulting in the dispersion of clutter energy and subsequent degradation in clutter suppression performance. In comparison, the VB-SR-STAP algorithm does not require the setting of regularization parameters, and it continuously updates Equation (18) to address quantization errors. Moreover, its recovered clutter spectrum does not show significant broadening. The clutter energy is concentrated on the clutter ridge, demonstrating its superiority in maintaining spectral integrity. The IVB-SR-STAP algorithm, by updating only a selected few key hyperparameters, is also able to produce a clutter spectrum that compares favorably with the performance of the VB-SR-STAP algorithm. Both the VB-SR-STAP and IVB-SR-STAP algorithms overcome the challenges posed by the setting of regularization parameters, and the results they produce are very close to the optimal clutter spectrum, highlighting their exceptional accuracy and efficiency.

#### 4.1.2. Clutter Power Spectrum Under Array Element Error Conditions

In this subsection, we consider the non-ideal scenario with gain-phase (GP) errors. GP errors arise from inconsistent amplitude and phase characteristics in the radio frequency (RF) amplifier components of the array channels. These errors manifest as variations in amplitude and phase across different channels. To describe this, we introduce an error matrix T into the steering vector modeling. By extending the signal model to account for GP errors, Equation (20) can be rewritten as follows:

(49) $$X_{\mathrm{GP}}=T\Phi Y+N_{0}$$

where error matrix T is:

(50) $$T=I_{M}⨂\mathrm{diag}([g_{1}\mathrm{exe}(jh_{1}),\cdots ,g_{N}\mathrm{exe}(jh_{N})])$$

where $g_{i}\in \left[0,0.1\right]$, $h_{i}\in [0,0.1\pi ]$, i = 1, …, N represents the amplitude error and phase error of i-th element.

**Figure 4:**
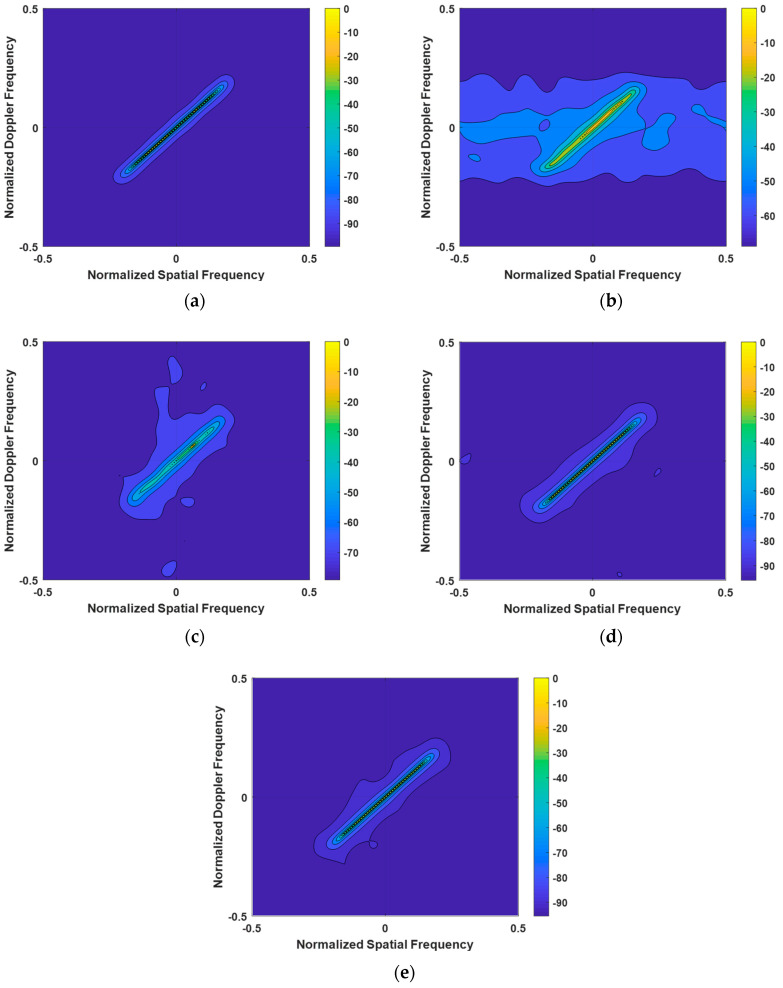
Clutter Power Spectrum under Array Element Error Conditions. (**a**) OPT; (**b**) LSSME-SR-STAP algorithm; (**c**) Homotopy-SR-STAP algorithm; (**d**) VB-SR-STAP algorithm; (**e**) IVB-SR-STAP algorithm.

When analyzing the clutter spectra of various algorithms shown in Figure 4 under the condition of GB errors, we can draw the following conclusions: The clutter spectrum calculated by the LSSME-SR-STAP algorithm exhibits significant broadening. This indicates that, under the influence of GB errors, the performance of this algorithm in clutter suppression is greatly affected, resulting in an increased width of the clutter spectrum. Compared to the VB-SR-STAP and IVB-SR-STAP algorithms, the clutter spectrum computed by the Homotopy-SR-STAP algorithm is slightly wider. This implies that, under the same GB error conditions, the Homotopy-SR-STAP algorithm is slightly inferior in maintaining the compactness of the clutter spectrum. The performance of the VB-SR-STAP and IVB-SR-STAP algorithms is noteworthy. The clutter spectra calculated by these two algorithms are close to the optimal clutter spectrum, demonstrating their excellent clutter suppression performance even in the presence of GB errors. This shows that the VB-SR-STAP and IVB-SR-STAP algorithms exhibit greater robustness and adaptability in addressing GB errors.

### 4.2. Analysis of Signal-to-Clutter-and-Noise Ratio Loss (SCNR Loss)

#### 4.2.1. Signal-to-Clutter-and-Noise Ratio Loss Under Ideal Conditions

The second experiment compares the SCNR Loss of the LMSSE-STAP, Homotopy-SR-STAP, VB-SR-STAP, and IVB-SR-STAP algorithms to evaluate their clutter suppression performance. The SCNR Loss is calculated as follows:

(51) $${\mathrm{SCNR}}_{\mathrm{Loss}}=\frac{\sigma_{n}^{2}{\left|W_{\mathrm{OPT}}^{H}V_{t}\right|}^{2}}{\mathrm{NM}W_{\mathrm{OPT}}^{H}R_{C}W_{\mathrm{OPT}}}$$

In the equation, $\sigma_{n}^{2}$ represents the noise power.

**Figure 5:**
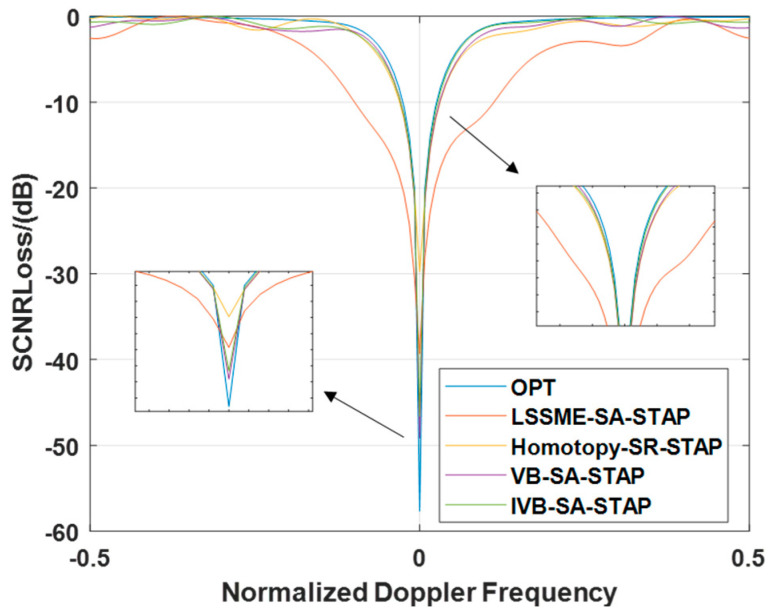
Signal-to-Clutter-and-Noise Ratio Loss under Ideal Conditions.

In ideal scenarios, the experimental results presented in Figure 5 reveal a compelling discovery: The SCNR Loss curve of the LMSSE-SR-STAP algorithm exhibits the most prominent notch width. Compared to the VB-SR-STAP algorithm and the IVB-SR-STAP algorithm proposed in this paper, the SCNR loss curve of the Homotopy-SR-STAP algorithm also displays a relatively wide notch, albeit less pronounced than that of the LMSSE-SR-STAP algorithm. Within the mainlobe clutter region, both the IVB-SR-STAP algorithm and the VB-SR-STAP algorithm are able to form deeper notches compared to the LMSSE-SR-STAP algorithm and the Homotopy-SR-STAP algorithm, with both trailing closely behind the optimal performance. It is worth emphasizing that the proposed new algorithm demonstrates its exceptional effectiveness and remarkable clutter suppression capabilities while significantly reducing the computational burden.

#### 4.2.2. Signal-to-Clutter-and-Noise Ratio Loss Under Array Element Error Conditions

**Figure 6:**
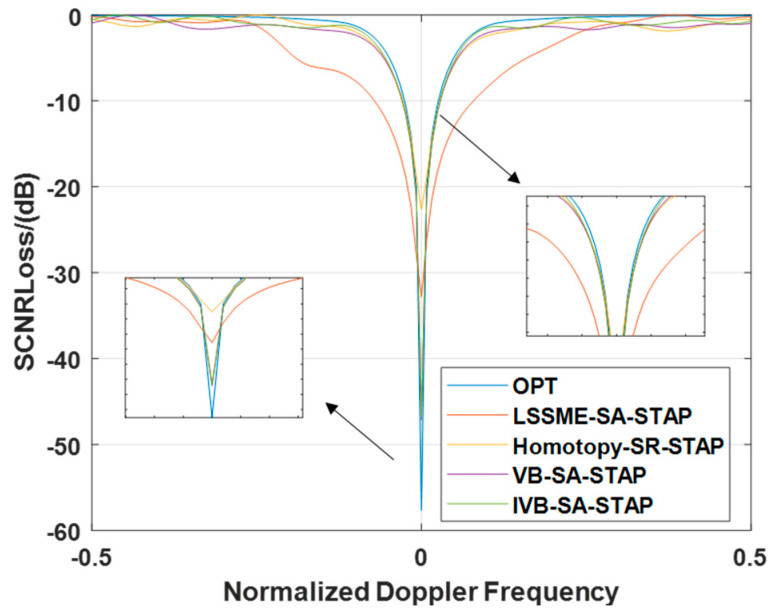
Signal-to-Clutter-and-Noise Ratio Loss under Array Element Error Conditions.

In this subsection, we delve into the specific performance of different algorithms in terms of SCNR Loss when faced with GB errors. By observing Figure 6, it is evident that the SCNR Loss notch produced by the LMSSE-SR-STAP algorithm is significantly broader than that of other algorithms. In contrast, although the Homotopy-SR-STAP algorithm is comparable to the VB-SR-STAP and IVB-SR-STAP algorithms in terms of the width of the SCNR loss notch, it falls significantly behind in the depth of the notch formed in the main clutter region, which is notably less than that of the VB-SR-STAP and IVB-SR-STAP algorithms. Based on the above analysis, we can conclude that in complex environments with array element errors, the IVB-SR-STAP algorithm proposed in this paper not only successfully reduces the computational complexity of the VB-SR-STAP algorithm but also maintains excellent clutter suppression performance. This discovery further validates the feasibility and advantages of the IVB-SR-STAP algorithm in practical applications.

## 5. Conclusions

In this paper, a hierarchical Bayesian prior framework is proposed, and iterative update formulas for parameters are derived through variational inference methods. Leveraging the prior information on the rank of the spatio-temporal clutter covariance matrix, an improved variational Bayesian approach is introduced, optimizing the updated formulas for parameters and effectively reducing computational complexity. Furthermore, this method fully exploits the joint sparsity of the multiple measurement vector model, achieving higher sparsity while maintaining high accuracy, and employs a first-order Taylor expansion to eliminate grid mismatch in the dictionary. Experimental results demonstrate that the proposed algorithm maintains excellent performance while effectively reducing computational complexity, showcasing its remarkable efficiency and effectiveness.

## Figures and Tables

**Table 1:** Simulation parameters of the radar system with a side-looking uniform linear array.

Parameters	Value
Number of Array Elements	10
Number of Pulses	10
Element Spacing (m)	0.1
Operating Wavelength (m)	0.2
Flight Speed (m/s)	150
Flight Altitude (m)	4000
Pulse Repetition Frequency (Hz)	5000
$\rho_{d}$	4
$\rho_{s}$	4
Training snapshot number	10
SNR (dB)	30

## Data Availability

Restrictions apply to the datasets.
